# Specific Hsp100 Chaperones Determine the Fate of the First Enzyme of the Plastidial Isoprenoid Pathway for Either Refolding or Degradation by the Stromal Clp Protease in Arabidopsis

**DOI:** 10.1371/journal.pgen.1005824

**Published:** 2016-01-27

**Authors:** Pablo Pulido, Ernesto Llamas, Briardo Llorente, Salvador Ventura, Louwrance P. Wright, Manuel Rodríguez-Concepción

**Affiliations:** 1 Centre for Research in Agricultural Genomics (CRAG) CSIC-IRTA-UAB-UB, Campus UAB Bellaterra, Barcelona, Spain; 2 Institut de Biotecnologia i Biomedicina and Departament de Bioquímica i Biologia Molecular, Universitat Autònoma de Barcelona, Campus UAB Bellaterra, Barcelona, Spain; 3 Max Planck Institute for Chemical Ecology, Jena, Germany; University of Massachusetts at Amherst, UNITED STATES

## Abstract

The lifespan and activity of proteins depend on protein quality control systems formed by chaperones and proteases that ensure correct protein folding and prevent the formation of toxic aggregates. We previously found that the *Arabidopsis thaliana* J-protein J20 delivers inactive (misfolded) forms of the plastidial enzyme deoxyxylulose 5-phosphate synthase (DXS) to the Hsp70 chaperone for either proper folding or degradation. Here we show that the fate of Hsp70-bound DXS depends on pathways involving specific Hsp100 chaperones. Analysis of individual mutants for the four Hsp100 chaperones present in Arabidopsis chloroplasts showed increased levels of DXS proteins (but not transcripts) only in those defective in ClpC1 or ClpB3. However, the accumulated enzyme was active in the *clpc1* mutant but inactive in *clpb3* plants. Genetic evidence indicated that ClpC chaperones might be required for the unfolding of J20-delivered DXS protein coupled to degradation by the Clp protease. By contrast, biochemical and genetic approaches confirmed that Hsp70 and ClpB3 chaperones interact to collaborate in the refolding and activation of DXS. We conclude that specific J-proteins and Hsp100 chaperones act together with Hsp70 to recognize and deliver DXS to either reactivation (via ClpB3) or removal (via ClpC1) depending on the physiological status of the plastid.

## Author Summary

In this paper we report a relatively simple mechanism by which plant chloroplasts deal with inactive forms of DXS, the main rate-determining enzyme for the production of plastidial isoprenoids relevant for photosynthesis and development. We provide evidence supporting that particular members of the Hsp100 chaperone family contribute to either refold or degrade inactive DXS proteins specifically recognized by the J-protein adaptor J20 and delivered to Hsp70 chaperones. Our results also unveil a J-protein-based mechanism for substrate delivery to the Clp complex, the main protease in the chloroplast stroma. Together, this work allows a better understanding of how chloroplasts get rid of damaged DXS (and potentially other proteins), which should contribute to take more informed decisions in future approaches aimed to manipulate the levels of plastidial metabolites of interest (including vitamins, biofuels, or drugs against cancer and malaria) in crop plants.

## Introduction

Organelles like mitochondria and plastids play fundamental roles in all eukaryotic organisms. In particular, plastids were acquired by a symbiosis between photosynthetic cyanobacteria and eukaryotic cells. Today, plastids (like mitochondria) are intimately integrated into the metabolism of plant cells but they still remain as separate functional entities that regulate their own biochemistry by relatively independent mechanisms. An important part of this regulation relies on the effective control of plastidial enzyme activities. Most of the enzymes required for plastidial metabolism are encoded by nuclear genes, synthesized in precursor form in the cytosol, and transported into plastids using energy-dependent import machineries [1]. Following import, specific proteases cleave the transit peptides and complex networks of plastidial chaperones ensure proper folding, assembly, or suborganellar targeting of the mature proteins. Chaperones and proteases are also essential components of the protein quality control (PQC) system that promotes the stabilization, refolding, or degradation of mature proteins that lose their native conformation and activity after metabolic perturbations or environmental challenges such as excess light, temperature peaks, oxidative stress or nutrient starvation [2,3]. While plant plastids contain many groups of prokaryotic-like chaperones (such as Hsp70 and Hsp100) and proteases (including Clp, Lon, Deg, and FstH), their specific targets and PQC-related roles remain little studied [1–4].

Due to the presence of plastids, plants have biochemical pathways that are not found in other eukaryotic kingdoms. For example, isoprenoid precursors are produced by the methylerythritol 4-phosphate (MEP) pathway in bacteria and plant plastids, whereas animals and fungi synthesize these essential metabolites using a completely unrelated pathway which is also used by plants to produce cytosolic and mitochondrial isoprenoids [5,6]. MEP-derived isoprenoids include compounds essential for photosynthesis (such as carotenoids and the side chain of chlorophylls, tocopherols, plastoquinone and phylloquinones) and growth regulation (including the hormones gibberellins, cytokinins, strigolactones and abscisic acid). Many plastidial isoprenoids also have nutritional and economic relevance [6]. All MEP pathway enzymes are located in the plastid stroma [5,7]. While transcriptional regulation of genes encoding biosynthetic enzymes is known to exert a coarse control of the MEP pathway, fine-tuning of metabolic flux appears to rely on post-transcriptional or/and post-translational regulation of enzyme levels and activity [8–12]. This is most evident for deoxyxylulose 5-phosphate synthase (DXS), the homodimeric enzyme that catalyzes the first step of the pathway. Metabolic control analysis calculations confirmed that DXS is the enzyme with the highest flux control coefficient (i.e. the main rate-determining step) of the MEP pathway [13]. Consistent with this prime regulatory role, DXS activity is tightly regulated by several post-translational mechanisms [10–12]. In particular, DXS enzymatic activity is allosterically inhibited by MEP pathway products [14,15], which also repress DXS protein accumulation [8,14,16–18]. Mathematical modeling recently showed that the post-translational control of DXS protein abundance and enzyme activity is crucial for the adjustment of the MEP pathway flux to persistent changes in environmental conditions, such as substrate supply or product demand [18]. Despite the central relevance of this type of regulation, little is known about the molecular mechanisms behind it.

We previously showed that the *Arabidopsis thaliana* J-protein J20 interacts with inactive forms of DXS to deliver them to the Hsp70 chaperone for eventual activation (which involves folding or refolding) or degradation (which involves unfolding) [19]. However, the particular protease involved and the specific components of the two J20-dependent antagonistic pathways remained unknown. Here we show that DXS is primarily degraded by the Clp protease complex through a pathway involving J20 and Hsp100 chaperones of the ClpC type. We also demonstrate that Hsp70 can physically interact with ClpB3, another plastidial Hsp100 chaperone, to promote the activation of non-functional DXS enzymes.

## Results and Discussion

### DXS appears to be primarily degraded by the stromal Clp protease

The main protease families involved in the degradation of terminally damaged or surplus proteins in plastids are Clp, Lon, Deg, and FtsH, all of them of prokaryotic origin [3,4]. We and others have previously shown that Arabidopsis mutants with a decreased activity of the stromal Clp protease complex display an accumulation of several MEP pathway enzymes, including DXS [20–24]. However, whether other plastidial proteases involved in PQC networks could also contribute to DXS degradation in the stroma remains unexplored. Several functional Lon homologues are found in Arabidopsis, but only Lon1 [25] and Lon4 [26] have been localized to chloroplasts, where they are attached to the stromal side of thylakoids. The Deg gene family in Arabidopsis contains 16 members, with 5 of them experimentally confirmed to be localized in chloroplasts [27]. From these, the isoforms Deg1, Deg5 and Deg8 were found in the thylakoid lumen, whereas Deg2 and Deg7 were detected in the stromal side [28,29]. FtsH proteases are encoded by 12 genes in Arabidopsis, and 9 of them can be found in chloroplasts [30]. The four major chloroplast isomers (FtsH2, FtsH5, FtsH8 and FtsH1, in order of abundance) have been shown to reside in the thylakoid membrane with their catalytic domain facing the stromal side [31–33].

**Fig 1:**
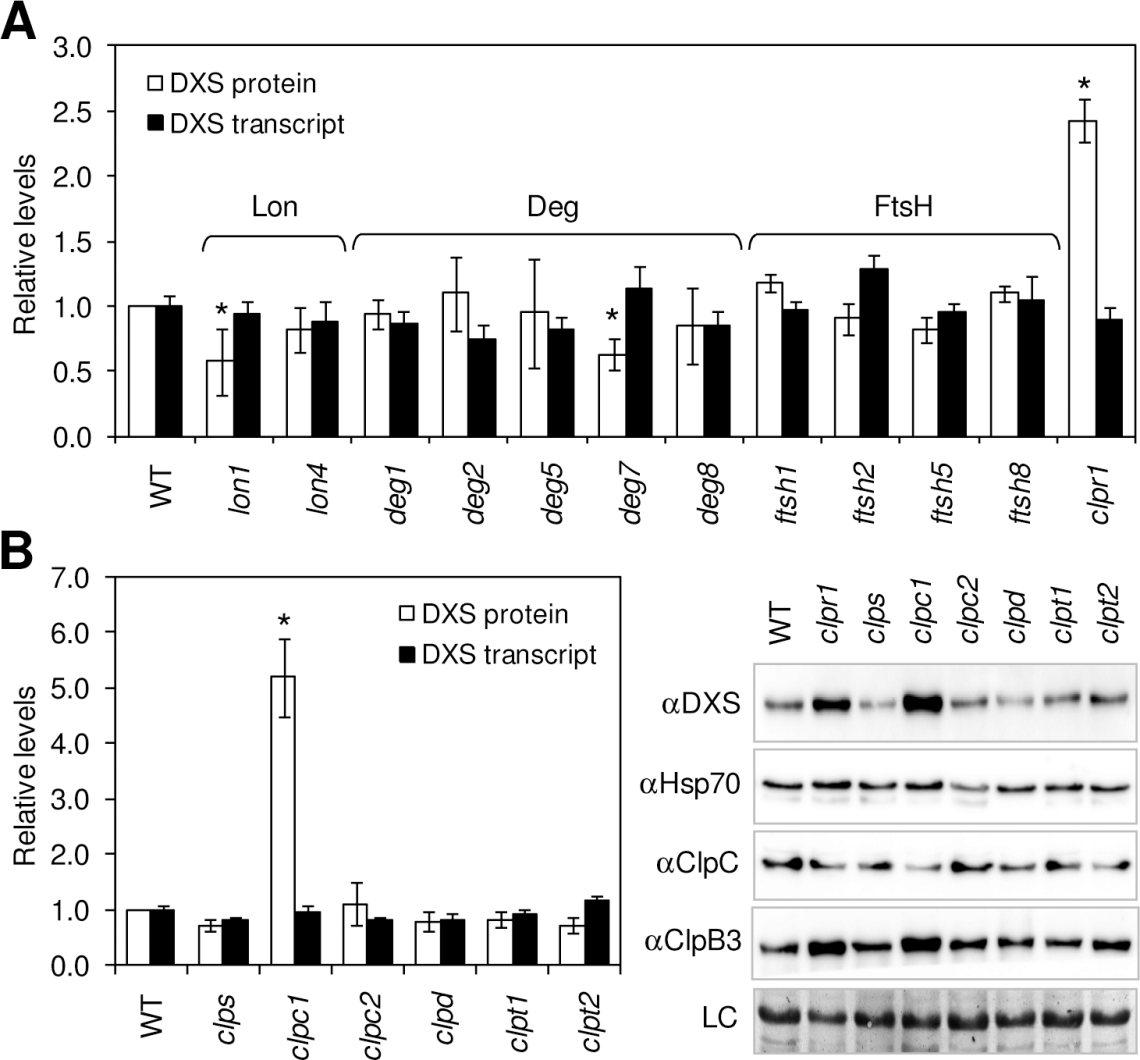
DXS is primarily degraded by the Clp protease. Columns represent DXS protein and transcript levels in 10-day-old WT plants and single mutants defective in the indicated subunits of plastidial proteases (A) or regulatory components of the Clp protease complex (B). Data correspond to the mean and SEM values of n≥3 independent experiments and are represented relative to the levels in WT plants. Asterisks mark statistically significant differences (*t* test: p<0.05) relative to WT samples. Representative images of immunoblot analyses with the indicated antibodies and a Coomassie blue staining of the blots (loading control, LC) are also shown in (B).

DXS protein levels were examined by immunoblot analysis in Arabidopsis wild-type (WT) plants and single mutants defective in plastidial Lon, Deg, or FtsH isomers (Fig 1 and S1 Table). As a control, we included the Clp protease mutant *clpr1*, which displays a reduction of other subunits of the Clp proteolytic core [34] but increased DXS protein levels [20]. As shown in Fig 1A, DXS protein levels in the analyzed mutants were similar to those in WT plants with only three exceptions. Lines defective in Lon1 and Deg7 showed a decreased accumulation of the protein compared to the WT, whereas DXS levels were only increased in the *clpr1* mutant (Fig 1A). No changes in *DXS* transcript levels were detected in any of the mutant lines (Fig 1A). Since enzyme levels would be expected to be post-translationally increased (but not decreased) in the mutants impaired in DXS-degrading proteases, we conclude that the Clp complex is likely the primary protease for DXS removal. The contribution of Lon, Deg, or FtsH proteases, however, cannot be fully discarded as we only tested mutants for individual isoforms of those other proteases and it is possible that different isoforms may have redundant functions.

### ClpC chaperones are required for the degradation of DXS by the Clp protease

Clp proteases are found in almost all bacteria and endosymbiotic organelles (mitochondria and plastids). In bacteria (S1 Fig), they are formed by a barrel-like catalytic core of two heptameric rings of proteolytic subunits (ClpP) and a dynamically interacting hexameric ring of Hsp100 chaperones (ClpA and ClpX in *Escherichia coli*; ClpC, ClpX, and ClpE in *Bacillus subtilis*) that unfold substrates for translocation into the proteolytic chamber [35]. Additionally, interaction of Hsp100 members with adaptor proteins (such as ClpS and SspB in *E*. *coli* and ClpS, MecA, McsB, and others in *B*. *subtilis*) enhance or expand substrate specificity [35–37]. In plant plastids, the Clp protease is more complex [38,39] but the basic components are conserved (S1 Fig). It presents a protease core (formed by two heptameric rings of plastome encoded ClpP1 and nuclear-encoded ClpP3-P6 and ClpR1-R4 subunits) stabilized by two plant-specific subunits (ClpT1-T2). The Arabidopsis homologues of the bacterial ClpA and ClpC unfolding chaperones are ClpC1, ClpC2, and ClpD. A ClpS adaptor is also found in chloroplasts [40], where it might form a plant-specific binary adaptor complex with the ClpF protein [41]. The possibility of other pathways delivering proteins to the Clp protease, however, remains open.

As shown above (Fig 1A), DXS levels increase in mutants defective in Clp protease activity such as *clpr1* [20]. If DXS is targeted to the Clp protease for degradation, we would also expect a post-translational upregulation of DXS enzyme levels in mutants impaired in the adaptors and chaperones that deliver the protein to the Clp catalytic core. A systematic analysis of such mutants (*clps*, *clpc1*, *clpc2*, *clpd*, *clpt1*, and *clpt2*) showed that only those defective in ClpC1 accumulated higher levels of DXS protein than WT plants (Fig 1B and S2 Fig). Quantification of DXS-encoding transcripts in the same mutant lines showed WT levels in all cases (Fig 1B), confirming that the observed accumulation of DXS polypeptides in ClpC1-defective lines was not a consequence of increased gene expression.

**Fig 2:**
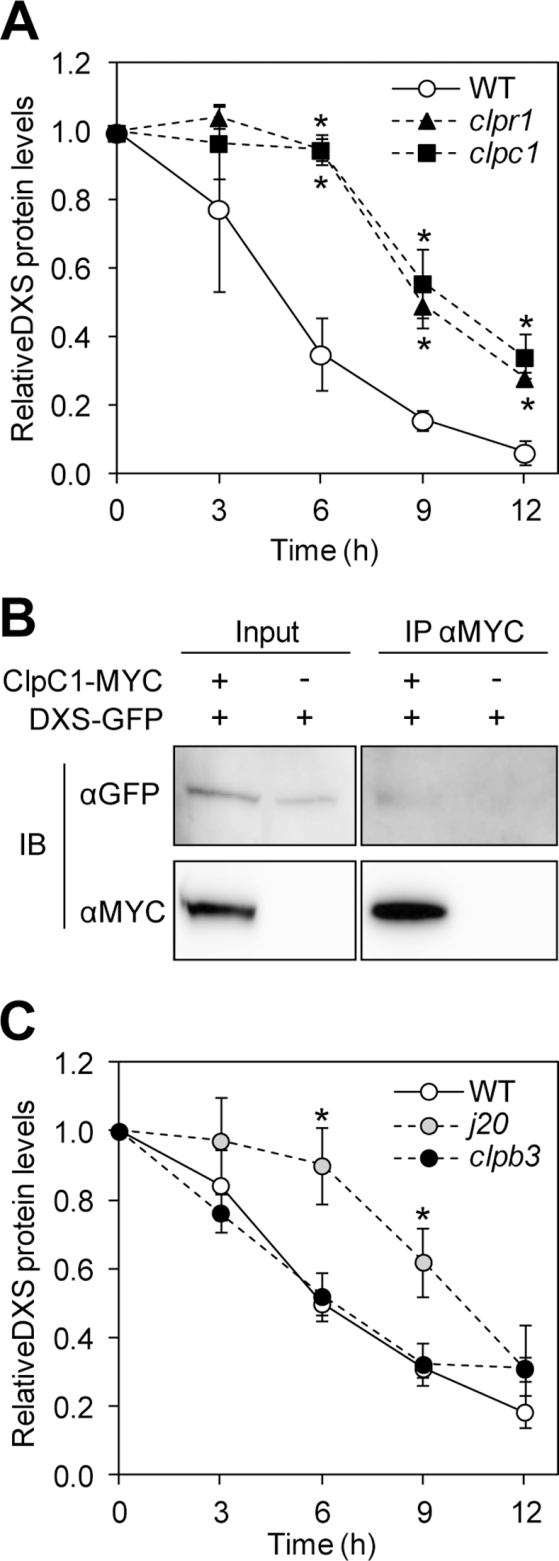
J20 and ClpC1 are required for normal DXS degradation. (A) WT plants and mutants defective in ClpR1 or ClpC1 were grown for one week on medium lacking the protein synthesis inhibitor cycloheximide and then treated with the inhibitor for the indicated times. DXS protein levels detected by immunoblot analysis are represented relative to those before treatment. (B) Protein extracts from *Nicotiana benthamiana* plants transiently producing DXS-GFP alone or together with a MYC-tagged ClpC1 protein were used for immunoprecipitation (IP) with anti-MYC antibodies (αMYC) and further immunoblot (IB) analysis with anti-GFP or anti-MYC sera. Immunoblot analyses of the extracts before immunoprecipitation (Input samples) are also shown. (C) Protein degradation rates of DXS in WT plants and mutants defective in J20 or ClpB3. The experiment was performed as described in (A). Mean and SEM of n≥3 experiments are shown in (A) and (C). Asterisks mark statistically significant differences (*t* test: p<0.05) relative to WT samples.

It has been proposed that the two Arabidopsis ClpC paralogs ClpC1 and ClpC2 perform similar if not identical functions in the chloroplast [42]. However, proteolytic assays with known Clp protease substrates only showed a greatly reduced degradation rate in *clpc1* plants [42], which showed the strongest reduction in total ClpC content (Fig 1B and S2 Fig). Estimation of DXS degradation rates upon treating WT and mutant plants with the protein synthesis inhibitor cycloheximide also showed a slower proteolytic removal of DXS polypeptides in *clpc1* mutants (Fig 2A). As expected, a defective Clp catalytic core in the *clpr1* mutant led to similarly reduced DXS degradation rates (Fig 2A), again supporting our conclusion that DXS is a target for this proteolytic complex. To confirm whether DXS might be a ClpC1 substrate, tagged versions of the Arabidopsis proteins (DXS-GFP and ClpC1-MYC) were overproduced in *Nicotiana benthamiana* leaves by agroinfiltration and co-immunoprecipitation assays were next performed. As shown in Fig 2B, these assays confirmed that DXS and ClpC1 can indeed interact. Together, we conclude that DXS might be mainly unfolded by ClpC1 for degradation by the Clp proteolytic core.

### J20 together with Hsp70 might deliver DXS to ClpC chaperones for eventual degradation

Recent results have shown that client proteins of the stromal Clp protease are recognized and delivered to ClpC chaperones by ClpS and ClpF adaptors [40,41]. While DXS might actually be a target of ClpS in bacteria [43], a wild-type phenotype in terms of DXS protein levels was observed in Arabidopsis plants defective in the proposed chloroplast adaptors (Fig 1B) [40,41]. Although ClpC could possibly directly deliver client proteins to the Clp protease without the need of an adaptor, we reasoned that further substrate specificity should be achieved using an alternative ClpS/ClpF-independent adaptor system.

Our previous work showed that inactive forms of DXS are recognized by J20, a J-protein adaptor that delivers them to the Hsp70 chaperone [19]. Computational analysis of the Arabidopsis DXS monomer with the Aggrescan3D algorithm revealed the presence of several aggregation-prone clusters (S3 Fig). Consistent with the conclusion that DXS tends to aggregate and that J20 prevents its aggregation, GFP-tagged DXS proteins accumulate in plastidial speckles that are larger in *j20* plants (S4 Fig) [19]. In addition, the endogenous DXS enzymes are less accessible to proteinase K cleavage in the *j20* mutant (S4 Fig), again suggesting that DXS aggregation is increased in the absence of J20, likely because the delivery of aggregated (and hence inactive) DXS proteins to the Hsp70 chaperone is impaired. The main role of Hsp70 is actually to prevent the formation of toxic aggregates of damaged proteins and, together with Hsp100 chaperones, promote their solubilization [44–50]. However, Hsp70 chaperones also facilitate the transfer of irreparably damaged client proteins to proteolytic systems [49,51–53]. For example, cytosolic Hsp70 is involved in the degradation of Arabidopsis protein clients by the eukaryotic 26S proteasome [51].

**Fig 3:**
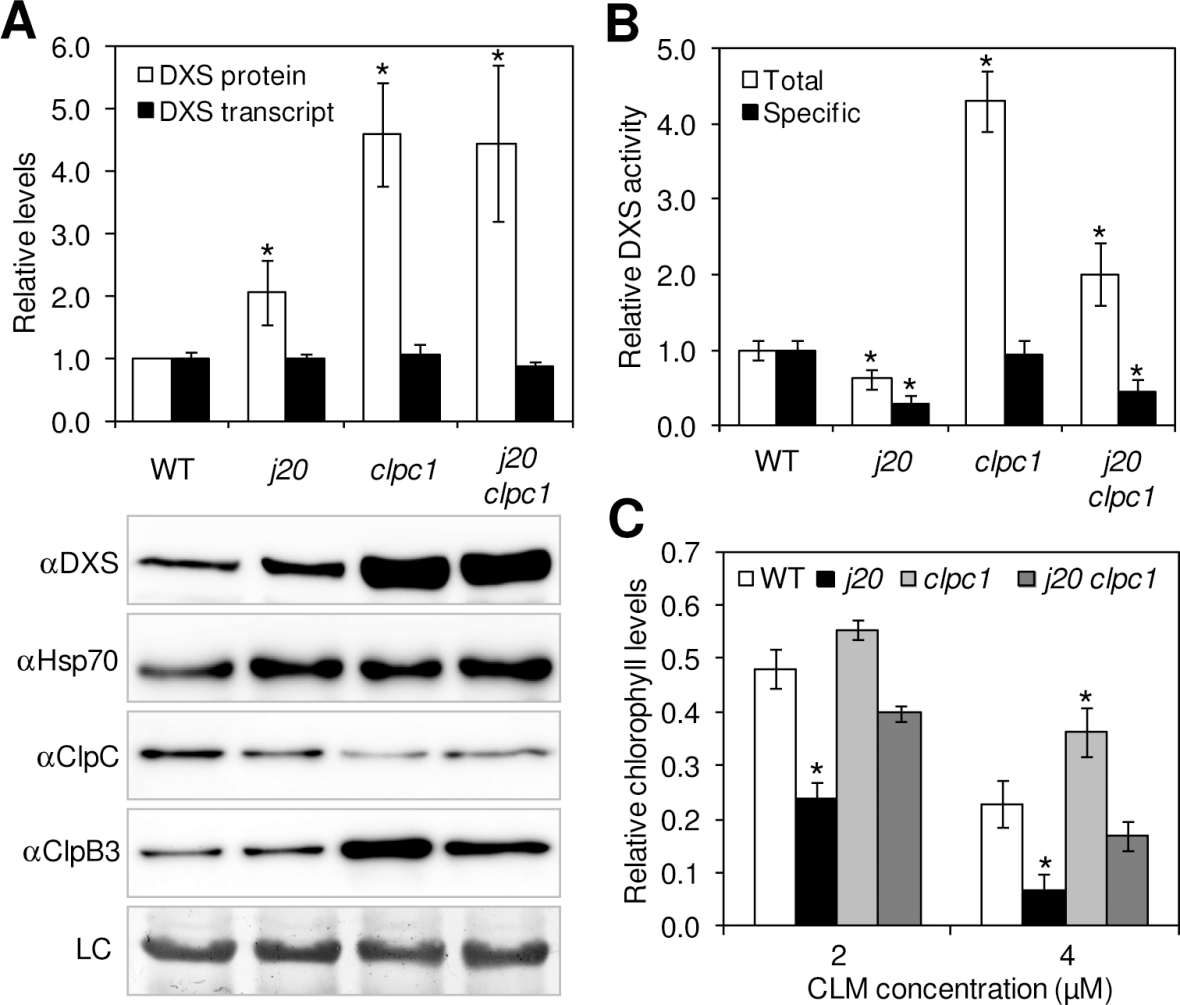
J20 and ClpC1 participate in the same pathway for DXS degradation. (A) Quantification of DXS protein and transcript levels in 10-day-old WT and mutant plants defective in J20, ClpC1, or both. Representative images of immunoblot analyses with the indicated antibodies and a loading control are also shown. (B) DXS activity levels in the indicated lines represented as total or specific (i.e. relative to the amount of DXS protein) values. (C) CLM resistance of the lines shown represented as the amount of chlorophyll remaining in plants grown at the indicated concentrations of the inhibitor. Levels are represented relative to those in WT plants in (A) and (B) or to untreated samples in (C). Data correspond to the mean and SEM values of n≥3 independent experiments and asterisks mark statistically significant differences (*t* test: p<0.05) relative to WT samples.

Despite the absence of conserved domains for direct interactions between Hsp70 and ClpC-type Hsp100 proteins (S5 Fig) [36,45,46], co-immunoprecipitation experiments showed that both chaperones can be found together in the chloroplast envelope [54,55]. It is therefore possible that Hsp70 and ClpC might interact either directly (using unidentified chaperone binding motifs) or indirectly (via third partners) to participate in PQC events at the stromal side of the inner envelope membrane [1,42,56,57]. Because in Arabidopsis the two plastidial isoforms of Hsp70 (Hsp70.1 and Hsp70.2) and ClpC (ClpC1 and ClpC2) are also found in the stroma [42,58], we reasoned that Hsp70 and ClpC proteins might collaborate to deliver DXS to the Clp protease using J20 as an adaptor. Interestingly, overexpression of J20 in transgenic Arabidopsis plants leads to decreased DXS protein levels, whereas loss of J20 function causes a reduced degradation rate of the enzyme (Fig 2C) [19]. Since both the J20 adaptor and ClpC chaperones are involved in the control of DXS degradation, we next tested whether they might function in the same pathway. We followed a genetic strategy based on comparing the DXS accumulation phenotype of single mutants defective in either J20 or ClpC1 with that of double *j20 clpc1* mutants (Fig 3). All three mutants accumulated higher levels of DXS proteins (but not transcripts) compared to WT plants. In particular, DXS levels increased ca. 2-fold in *j20* plants and 4-fold in single *clpc1* and double *j20 clpc1* mutants (Fig 3A). The absence of an additive or synergistic phenotype in the double mutant supports the conclusion that J20 and ClpC1 actually function in the same pathway delivering DXS to degradation in Arabidopsis plastids. Such a ClpS/ClpF-independent pathway could potentially be functioning for other plastidial clients of J-proteins. However, the lack of bona-fide substrates for other plastidial J-proteins prevents to experimentally testing this possibility at the moment.

### ClpB3 contributes to activation of J20-delivered DXS proteins by interaction with plastidial Hsp70 chaperones

The results described above suggest that damaged DXS polypeptides might be recognized by J20 and then delivered to Hsp70 and ClpC chaperones for unfolding and degradation by the Clp proteolytic core. But unlike that observed for J20-defective mutants [19], DXS accumulates in a mostly active form in *clpc1* mutants (Fig 3B). Thus, measurement of DXS activity in plant extracts showed increased total activity but unchanged specific activity (i.e. relative to protein levels) in *clpc1* compared to WT controls (Fig 3B). Estimation of DXS activity *in planta* by quantification of the resistance to clomazone (CLM), a specific DXS inhibitor [19,59,60] further supported the presence of higher DXS activity levels (i.e. increased resistance to the inhibitor) in the *clpc1* mutant, opposite to the increased sensitivity detected in the case of *j20* plants (Fig 3C and S6 Fig). To reconcile these results, we propose that loss of J20 causes an accumulation of aggregated (i.e. enzymatically inactive) DXS because the protein cannot be normally reactivated (refolded) or discarded (degraded). By contrast, accumulation of active DXS enzyme when ClpC activity decreases might be due to the existence of a functional pathway to disaggregate and refold the excess protein that cannot be degraded in the *clpc1* mutant. The participation of J20 in such a putative reactivation pathway is supported by the observation that loss of J20 function in degradation-impaired (ClpC1-defective) *j20 clpc1* plants results in a higher proportion of inactive DXS enzyme (Fig 3B) and hence a reduced resistance to CLM (Fig 3C and S6 Fig) compared to the single *clpc1* mutant.

**Fig 4:**
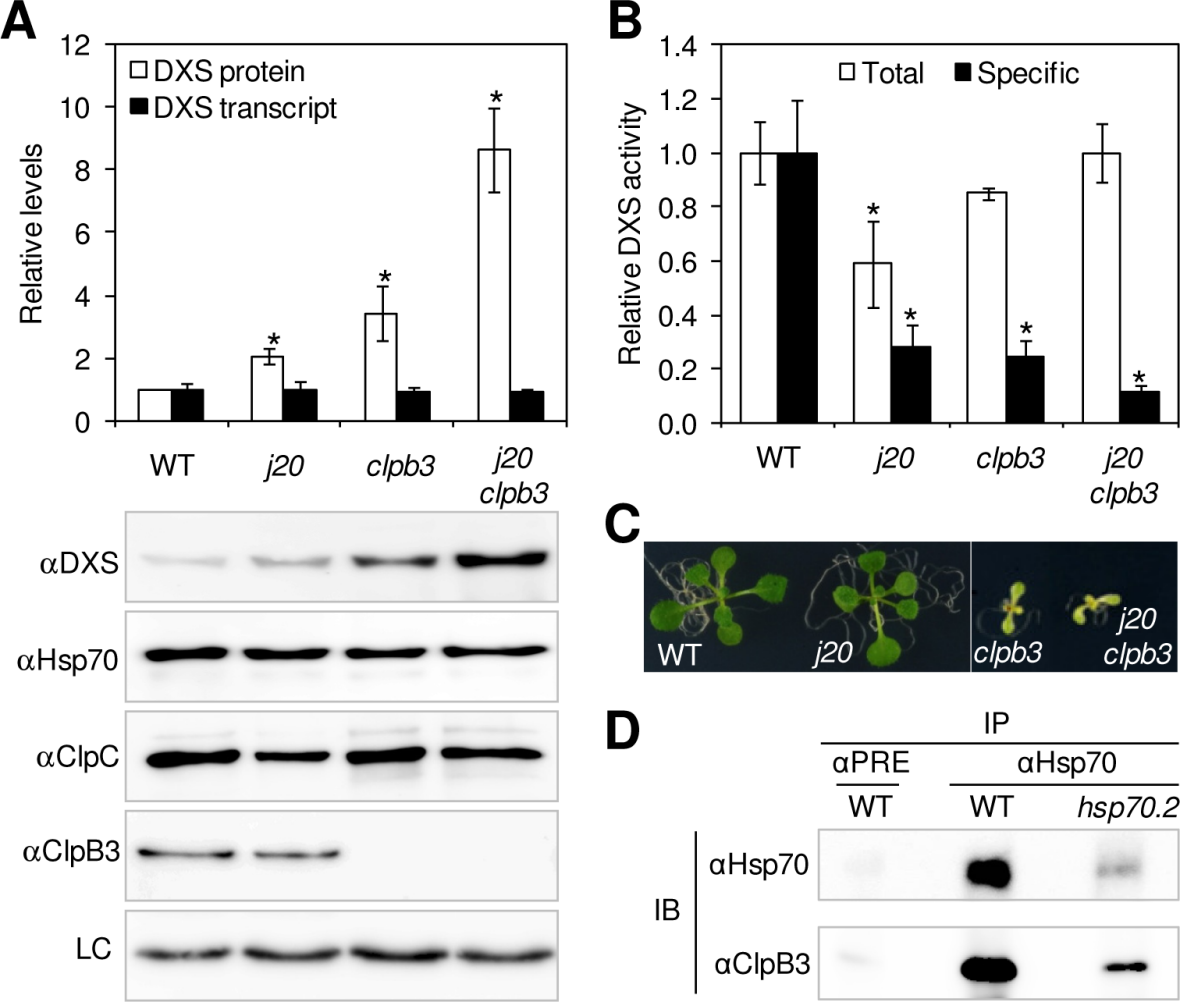
J20, Hsp70 and ClpB3 participate in the same pathway for DXS reactivation. (A) Quantification of DXS protein and transcript levels in 10-day-old WT and mutant plants defective in J20, ClpB3, or both. Representative images of immunoblot analyses with the indicated antibodies and a loading control are also shown. (B) DXS activity levels in the indicated lines represented as total or specific (i.e. relative to the amount of protein) values. Levels in (A) and (B) are represented relative to those in WT plants and correspond to the mean and SEM values of n≥3 independent experiments. Asterisks mark statistically significant differences (*t* test: p<0.05) relative to WT samples. (C) Representative picture of individual 10-day-old plants of the indicated lines. (D) Immunoprecipitation of ClpB3 with anti-Hsp70 antibodies. Protein extracts from WT or Hsp70.2-defective plants were used for immunoprecipitation (IP) with preimmune serum (αPRE) or an anti-Hsp70 antibody (αHsp70) and further immunoblot (IB) analysis with anti-Hsp70 (as a control) or anti-ClpB3 sera.

Work in different systems has shown that Hsp70 can be assisted by Hsp100 chaperones of the ClpB type in the solubilization of toxic aggregates of damaged proteins [45–50,61–64]. ClpB3 is the only ClpB-type chaperone found in Arabidopsis plastids [44]. Unlike the rest of plastidial Hsp100 chaperones present in this plant (ClpC1, ClpC2, and ClpD), ClpB3 lacks the IGF motif (or ClpP-loop) required for interaction with proteolytic subunits of the Clp core but it harbors a domain responsible for the interaction with Hsp70 chaperones (S5 Fig) [36,45,46]. Interestingly, the levels of ClpB3 were increased in mutants defective in Clp protease subunits, including ClpC1 [21,22,24,40,65] (Figs 1B and 3A and S2 Fig), suggesting that ClpB3 might contribute to mitigate protein folding stress caused by a defective Clp protease activity. In agreement, impairment of both ClpB3 and Clp protease activity results in a seedling lethal phenotype [22]. Based on these data, we speculated that ClpB3 might also participate in the DXS reactivation pathway mediated by J20 and Hsp70 chaperones. To evaluate this possibility, we first analyzed DXS protein levels and activity in ClpB3-defective Arabidopsis plants (Fig 4). If ClpB3 promotes DXS protein disaggregation (and hence activation), it was expected that *clpb3* mutants would show a transcription-independent accumulation of inactive forms of DXS, assuming that the degradation rate of J20-delivered proteins would remain constant. Indeed, *clpb3* plants showed a WT rate of DXS degradation (Fig 2C) but an enhanced accumulation of DXS enzyme without changes in transcript levels (Fig 4A). Also as predicted by our model, the specific activity of the DXS protein found in the ClpB3-defective mutant was much lower than that measured in WT plants (Fig 4B). Loss of both ClpB3 and J20 activities in the double *j20 clpb3* mutant resulted in an even higher accumulation (Fig 4A) of mostly inactive DXS protein (Fig 4B), presumably because the absence of J20 prevents the targeting of non-functional enzymes to ClpC for eventual degradation by the Clp protease. The dramatic phenotype displayed by single *clpb3* and double *j20 clpb3* mutant plants (Fig 4C) [66] prevented the reliable quantification of their CLM resistance. In any case, the available data suggests that when the proteolytic degradation of inactive (e.g. aggregated) forms of DXS delivered to the Clp protease by J20 via ClpC is impaired (e.g. in *clpr1* and *clpc1* mutants), an increase in ClpB3 levels promotes the disaggregation and activation of the enzyme, eventually resulting in higher levels of enzymatically active DXS. When J20 activity is missing, however, inactive DXS forms cannot be properly reactivated via ClpB3 (as deduced from the similar levels of DXS protein but lower proportion of active enzyme found in the double *j20 clpc1* mutant compared to the single *clpc1* line; Fig 3) or degraded via ClpC (as deduced from the increased levels of inactive DXS protein present in double *j20 clpb3* plants compared to the single *clpb3* mutant; Fig 4).

As described above, the mechanistic basis for the collaboration between J20, Hsp70, and ClpC chaperones is currently unknown. However, the presence of a Hsp70-binding motif in the amino acid sequence of ClpB3 (S5 Fig) suggests that plastidial Hsp70 isoforms might be able to directly interact with ClpB3 to synergistically activate damaged DXS proteins recognized by the J20 adaptor. In agreement with this possibility, the ClpB3 protein was efficiently immunoprecipitated from WT extracts using an anti-Hsp70 serum (Fig 4D). When a similar experiment was performed with the Arabidopsis *hsp70*.*2* mutant, previously shown to contain lower amounts of plastidial Hsp70 proteins than the WT [58], the level of immunoprecipitated ClpB3 protein was concomitantly decreased (Fig 4D). These results confirm that plastidial Hsp70 isoforms can be found together with ClpB3 in Arabidopsis chloroplasts, providing a mechanistic frame for the observed collaboration between these two families of chaperones in the J20-mediated activation of DXS.

### The fate of aggregated DXS proteins recognized by J20 and delivered to Hsp70 likely depends on the relative abundance of particular Hsp100 chaperones

The results described above are consistent with a model involving the participation of ClpB3 and ClpC1 on opposite pathways resulting in either reactivation or degradation, respectively, of inactive DXS proteins recognized by the Hsp70 adaptor J20. Under normal growth conditions, the levels of ClpB3 transcripts and protein are lower than those of ClpC1 (S7 Fig) [42,67]. However, *ClpB3* transcript levels have been shown to strongly increase upon exposure to high temperatures [66,68,69] whereas virtually no changes in RNA or protein levels have been detected for ClpC1 or ClpC2 in response to heat or other types of stress, including cold, drought, salt, and oxidative stress [66,70]. The ratio between plastidial ClpB3 and ClpC1 chaperones (and hence the potential capacity to reactivate damaged or/and aggregated DXS polypeptides) could therefore increase when plants are challenged with at least some types of stress (S7 Fig).

**Fig 5:**
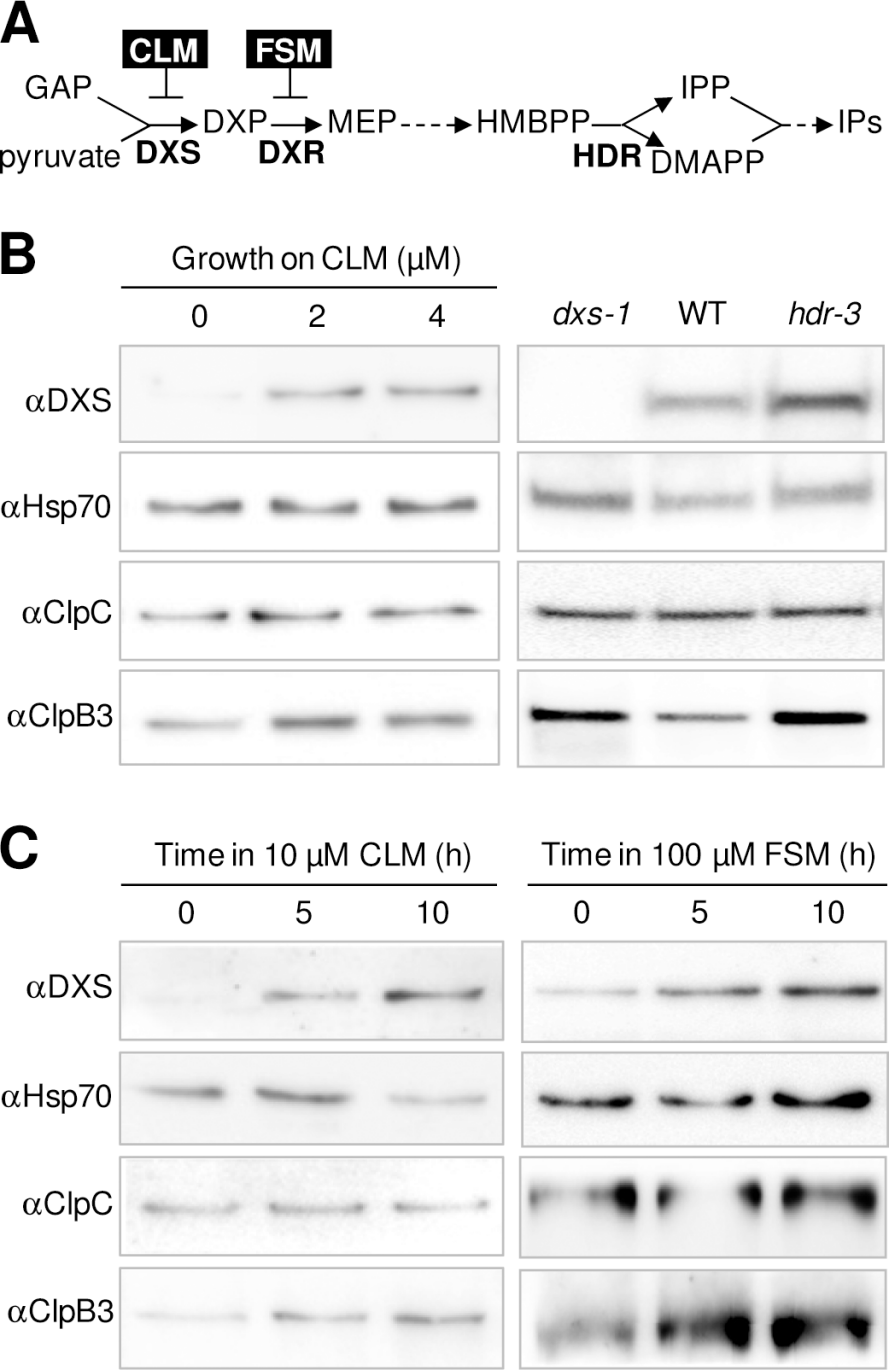
Accumulation of ClpB3 but not ClpC chaperones increases when DXS activity or MEP pathway flux decrease. (A) Schematic representation of the MEP pathway. GAP, glyceraldehyde 3-phosphate; DXP, deoxyxylulose 5-phosphate; MEP, methylerythritol 4-phosphate; HMBPP, hydroxymethylbutenyl diphosphate; IPP, isopentenyl diphosphate; DMAPP, dimethylallyl diphosphate; IPs, isoprenoids. Enzymes are represented in bold: DXS, DXP synthase; DXR, DXP reductoisomerase; HDR, HMBPP reductase. Inhibitors are boxed: CLM, clomazone; FSM, fosmidomycin. (B) Immunoblot analyses with the indicated antibodies of WT plants germinated and grown for 18 days on media supplemented with the indicated concentrations of CLM (left panels) or mutants defective in DXS (*dxs-1*) or HDR (*hdr-3*) grown for 10 days on media with no inhibitors (right panels). (C) Immunoblot analyses with the indicated antibodies of WT plants germinated and grown for 7 days on media with no inhibitors and then transferred for the indicated times to media supplemented with CLM (left panels) or FSM (right panels).

DXS-derived isoprenoids such as carotenoids and tocopherols protect plants against oxidative stress, whereas others (including chlorophylls and prenylated quinones) are essential for photosynthesis. Therefore, a decreased production of these isoprenoids (e.g. upon down-regulating DXS activity) is expected to trigger a stress response. We observed that a specific reduction in DXS activity in Arabidopsis WT plants germinated and grown in the presence of CLM caused an increased accumulation of ClpB3 but not ClpC chaperones compared to controls grown in the absence of inhibitor (Fig 5). A similar ClpB3 protein accumulation response was also observed in mutants with a defective MEP pathway (Fig 5). As previously observed [8,14,16–18], the pharmacological or genetic blockage of the pathway also resulted in increased accumulation of DXS protein. Most interestingly, the DXS and ClpB3 accumulation response was detected as soon as 5 hours after reducing the MEP pathway flux by treatment with specific inhibitors (Fig 5). We therefore conclude that stress situations (including those causing a decreased DXS activity and/or MEP pathway flux) could rapidly trigger an increased accumulation of ClpB3, but not ClpC chaperones, likely aimed to promote the reactivation pathway that would keep DXS enzymes in an enzymatically active condition. Furthermore, our data show that ClpB3 levels are more prone to change compared to those of ClpC proteins, suggesting that ClpB3 concentration might be a major factor regulating the fate of inactive DXS polypeptides recognized by J20 and delivered to Hsp70.

### A model for the role of J20, Hsp70, and Hsp100 chaperones in the regulation of DXS enzyme levels and activity in plastids

**Fig 6:**
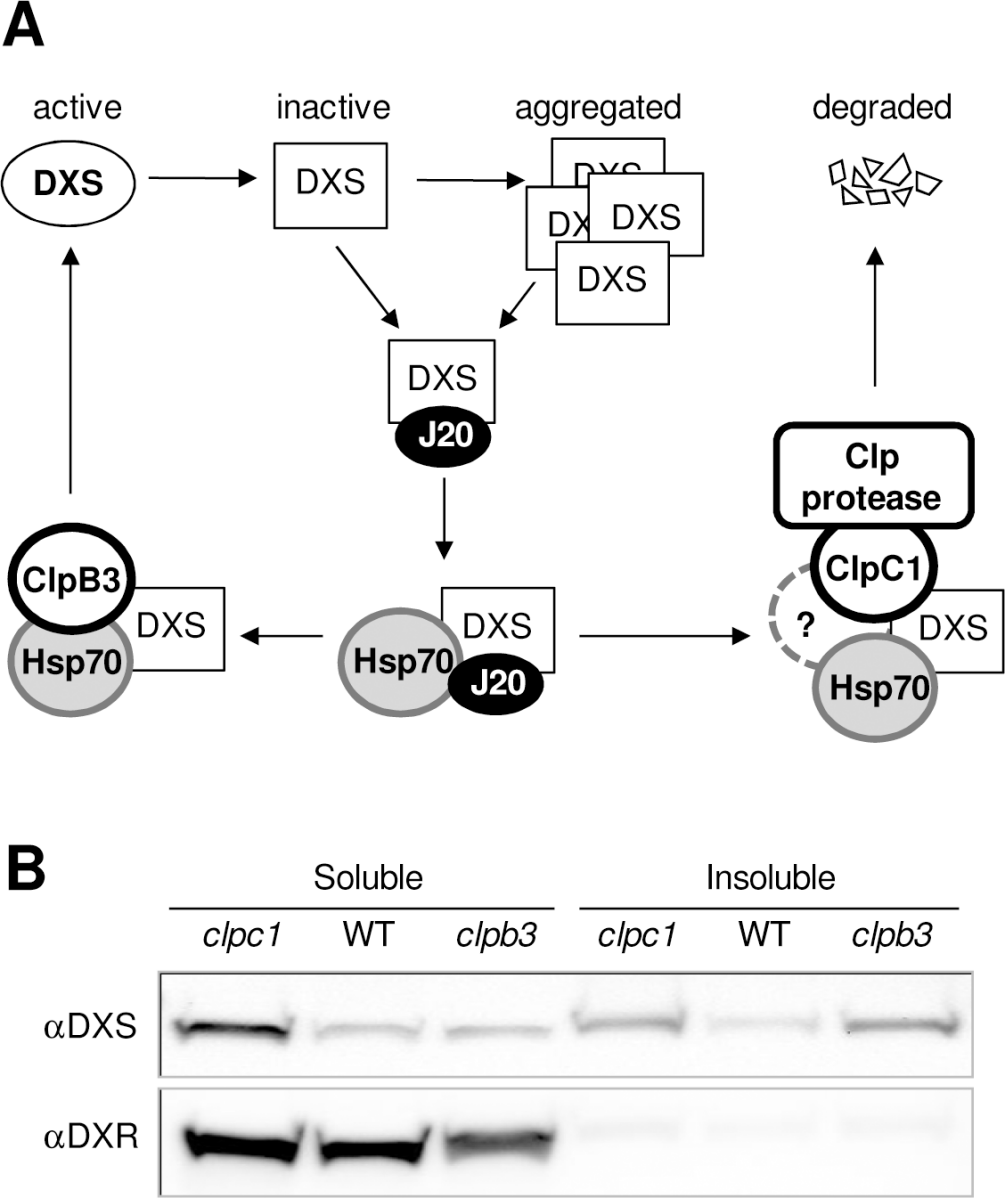
Model of the molecular pathways determining the fate of DXS in plastids. (A) The J-protein J20 facilitates the recognition of damaged (misfolded) or aggregated DXS proteins and their delivery to the Hsp70 chaperone. Then, interaction with the Hsp100 chaperone ClpB3 can synergistically contribute to refold the enzyme back to its active form. Alternatively, Hsp70 can deliver the inactive DXS protein to the Clp protease via ClpC1 for unfolding and degradation. (B) Distribution of DXS and DXR proteins in soluble and insoluble fractions in WT plants and mutants defective in any of the two Hsp100 chaperones that can determine the fate of J20-targeted DXS protein. Representative immunoblots are shown. Ratios of soluble vs. insoluble DXS protein levels (mean±SEM of at least 3 independent experiments) are as follows: WT, 1.84±0.05; *clpc1*, 1.99±0.26; *clpb3*, 0.36±0.08.

Based on the presented data, we propose a model for the regulation of DXS enzyme levels and activity by different types of plastidial chaperones (Fig 6). According to this model, J20 (a plastidial member of the J-domain protein family, also known as J-proteins or Hsp40 co-chaperones) acts as an adaptor providing substrate specificity [19]. In particular, J20 delivers inactive DXS proteins to Hsp70 chaperones that would next act together with particular Hsp100 proteins to either degrade (ClpC1) or reactivate (ClpB3) the enzyme (Fig 6A). J20 might recognize DXS polypeptides that remain unfolded after plastid import or become misfolded by ordinary perturbations and eventually aggregate (S3 and S4 Figs), a process that would render the protein more insoluble and enzymatically inactive. Under normal growth conditions, most DXS proteins remain soluble but some are indeed found associated to the insoluble fraction (Fig 6B). This might be due to the relative low levels of ClpB3 relative to ClpC1 (S7 Fig) [42,67]. In agreement, a further reduction in ClpB3 levels (e.g. in the *clpb3* mutant) results in a higher proportion of DXS protein associated to the insoluble fraction (Fig 6B) and hence inactive (Fig 4). By contrast, an enhanced accumulation of ClpB3 takes place in stress situations (Fig 5 and S7 Fig) or when Clp protease function is impaired (Figs 1B and 3A and S2 Fig) [21,22,24,40,65], likely aimed to mitigate general protein folding stress. In the case of DXS, a reduced degradation rate in the *clpc1* mutant (Fig 2A) results in increased levels of active (soluble) enzyme (Figs 3 and 6B) likely because a higher accumulation of ClpB3 prevents DXS aggregation. Similar to that proposed in other systems [47–50,61–64], ClpB3 directly interacts with Hsp70 to synergistically perform this role (Fig 4). The observed changes in DXS protein levels and solubility appear to be highly specific, as the next enzyme of the MEP pathway (Fig 5A), deoxyxylulose 5-phosphate reductoisomerase (DXR), was found to be essentially soluble in WT and Hsp100-defective mutants (Fig 6B) and to remain unchanged in J20-defective plants [19].

Interaction with CHIP, a co-chaperone that functions as an E3 ubiquitin ligase, converts Hsp70 from a protein-folding machine into a degradation factor that targets unfolded substrates for degradation by the eukaryotic 26S proteosome [51,71]. Based on genetic evidence (Fig 3) and published results that ClpC and Hsp70 chaperones can be found together in plastid complexes [54,55], we propose that Hsp70 and ClpC chaperones could somehow interact (either directly or by means of unidentified partners) to deliver client proteins like DXS to the Clp catalytic complex. In summary, our model (Fig 6A) proposes that collaboration of Hsp70 with Hsp100 chaperones might deliver inactive (misfolded or/and aggregated) forms of DXS (and potentially many other plastidial proteins recognized by specific J-proteins, the substrate adaptors for Hsp70) to either refolding (via ClpB3) or degradation (via ClpC chaperones). The seedling lethal phenotype of double mutants with no ClpB3 and Clp protease activity [22] illustrates the key relevance of these two seemingly antagonistic pathways for plant life. We speculate that taking a specific pathway (i.e. deciding whether to repair or degrade the protein) might depend on the relative abundance of these Hsp100 partners, particularly as a consequence of changes in ClpB3 levels. The main reason behind the existence of such sophisticated and expensive pathways for the regulation of DXS levels and activity is likely to be the major role demonstrated for this enzyme in the control of the MEP pathway flux [10,11,13]. Future work should next determine how the collaboration of different sets of plastidial chaperone types, and hence the fate of the client protein, is specifically regulated.

## Materials and Methods

### Plant material, treatments and constructs

*Arabidopsis thaliana* mutant lines used here are indicated in S1 Table (all in the Columbia background). Sibling lines expressing *35S*:*DXS-GFP* in WT and *j20* backgrounds were previously generated [19]. Seeds were surface-sterilized and germinated on solid Murashige and Skoog (MS) medium supplemented with 1% sucrose. Plants were grown under long day conditions as described [19]. For cycloheximide experiments, seeds were germinated on top of a sterile disc of synthetic fabric (SefarNitex 03-100/44). At day 7, the disc with the seedlings was transferred to fresh medium supplemented with 100 μM cycloheximide and samples were collected at different times afterwards (up to 12h) for immunoblot analysis. Inhibition of protein synthesis with cycloheximide had no visual effect on treated seedlings at the times used for the experiment (S8 Fig). Treatments with MEP pathway inhibitors were performed by transferring discs with 7-day-old seedlings to fresh medium supplemented with 10 μM clomazone (CLM) or 100 μM fosmidomycin (FSM). CLM resistance was estimated by quantifying chlorophyll levels in the presence of increasing concentrations of the inhibitor as described [60].

For transient expression and co-immunoprecipitation assays, an Arabidopsis full-length cDNA encoding ClpC1 without the stop codon was PCR-amplified, cloned into the pDONOR207 vector (Invitrogen), and subcloned into the Gateway vector pGWB417 to be expressed under the *35S* promoter with a C-terminal MYC epitope (*35S*:*ClpC1-MYC* construct). A *35S*:*DXS-GFP* construct was available in the lab [19]. Transient expression of these constructs was carried out by agroinfiltration of *Nicotiana benthamiana* leaves using the *Agrobacterium* GV3101 strain. Samples for immunoprecipitation were collected after 3 days.

### Protein extraction, immunoprecipitation and detection

Protein extracts were obtained from whole plants and used for immunoprecipitation assays or/and immunoblot analysis as described [19]. For the separation of soluble and insoluble (with protein aggregates) fractions, native protein extracts were obtained in a buffer containing 100 mM Tris-HCl pH7.9, 10 mM MgCl_2_, 1% (v/v) glycerol, and 20 μl/ml protease inhibitor cocktail (Sigma). After centrifugation for 10 min at 10.000 x*g*, the supernatant was collected as the soluble fraction. The pellet was washed with fresh buffer and centrifuged again. The obtained pellet fraction was then resuspended in denaturing TKMES buffer [19] and centrifuged again to collect the supernatant as the insoluble fraction. In all cases, protein concentration was determined using the Bio-Rad protein assay. For immunoblot assays, antibodies raised against DXS and DXR [19], GFP (Life Technologies), MYC (Millipore), and chloroplast Hsp70, ClpC, and ClpB proteins (Agrisera) were diluted 1:500 for DXS, 1:7,000 for DXR, 1:1,000 for GFP and MYC, 1:6,000 for Hsp70, 1:2,000 for ClpC, and 1:3,000 for ClpB. The total amount of protein loaded per lane was calculated for each particular antibody to remain in the linear range (S9 Fig). Chemiluminescent signals were visualized using a LAS-4000 (Fujifilm) image analyzer and quantified with Quantity One (Bio-Rad). Student´s *t* test was used to assess statistical significance of quantified differences.

### Protease accessibility assays

For protease accessibility assays, protein extracts from 10-day-old WT and *j20* seedlings containing 30 μg of total protein were incubated for 5 min at 37°C with increasing concentrations of Proteinase K (Invitrogen). After stopping the reaction with SDS-PAGE loading buffer, extracts were used for immunoblot analysis.

### DXS activity assays

DXS enzyme activity measurements were carried out as described [19,72]. Specific activity was calculated by dividing the total activity measured in extracts with the amount of DXS protein found in the corresponding sample.

### RNA isolation and qPCR analysis

RNA isolation, cDNA synthesis, and qPCR experiments were performed as described [19] using the *APT1* (At1g27450) gene for normalization.

### Prediction of aggregation propensity

The Aggrescan3D algorithm [73] was used to analyze protein aggregation propensity. Predictions were performed in static mode using a distance of aggregation analysis of 10 Å. The Arabidopsis DXS structure was modelled using Swiss-Model [74] on top of the 2.40 Å resolution *E*. *coli* DXS structure with PDB code 2O1S. Residues 72 to 707 of the Arabidopsis DXS monomer, sharing a sequence identity of 41.08% with the *E*. *coli* protein, were structurally aligned and modelled. The interface of the generated homodimer was evaluated with PDBePISA (http://www.ebi.ac.uk/pdbe/pisa/) rendering an area of 8892 Å and a predicted dissociation ΔG for the dimer of 51.2 kcal/mol (close to those of the template *E*. *coli* crystal structure, which exhibits an interface of 7970 Å and a dissociation ΔG of 59.1 kcal/mol).

## Supporting Information

S1 Fig Schematic models of the Clp protease complexes in bacteria and plant plastids. (PDF)

S2 Fig Immunoblot analyses of DXS, Hsp70 and Hsp100 chaperones in WT seedlings and mutant alleles defective in ClpC1 or ClpC2 (see S1 Table). LC, loading control.
(PDF)

S3 Fig Aggregation propensity of the Arabidopsis DXS monomer. The protein surface is colored according to Aggrescan3D score in gradient from red (high predicted aggregation propensity) to white (negligible impact on protein aggregation) to blue (high predicted solubility). The figure was generated with PyMOL using the aggregation propensities encoded in the temperature factor column of the A3D.pdb output.
(PDF)

S4 Fig Aggregation phenotypes of DXS in WT and *j20* plants. (A) Confocal microscopy analysis of GFP (green) and chlorophyll (red) fluorescence distribution within the chloroplast. Representative images obtained with the same confocal parameters from siblings harboring the same T-DNA insertion with the *35S*:*DXS-GFP* construct in a wild-type (WT) or J20-defective (*j20*) background are shown. (B) Analysis of DXS protein abundance by immunoblot analysis of protein extracts from WT and *j20* plants incubated with the indicated concentrations (μg/ml) of proteinase K. Representative blots and quantitative data corresponding to the mean and SE values of n = 4 independent experiments are shown. Asterisks mark statistically significant differences (*t* test: p<0.05) relative to WT samples.
(PDF)

S5 Fig Sequence alignment of plastidial Hsp100 chaperones. Alignment was performed using Clustal Omega (www.ebi.ac.uk/Tools/msa/clustalo) with protein sequences of *Arabidopsis thaliana* ClpD (At5g51070), ClpC1 (At5g50920), ClpC2 (At3g48870), and ClpB3 (At5g15450), *Bacillus subtilis* ClpC (AAA19233), and *Escherichia coli* ClpB (EDV64786). The generated alignment was then edited with Bioedit (http://www.mbio.ncsu.edu/bioedit/page2.html). The domain responsible for the interaction of *E*. *coli* ClpB with Hsp70 is boxed in blue. The tripeptide loop shown to be required for interaction with ClpP subunits of the Clp protease complex is boxed in red.
(PDF)

S6 Fig CLM resistance of lines defective in J20, ClpC1, or both. Representative pictures of plants of WT, single mutant, and double mutant lines germinated and grown for 10 days in the presence of the indicated concentrations of CLM are shown.
(PDF)

S7 Fig Comparison of transcript levels of genes encoding ClpC1 and ClpB3. Data were obtained from the Arabidopsis eFP browser at www.bar.utoront.ca and correspond to the gene expression map of Arabidopsis development (A) and abiotic stress treatments (B).
(PDF)

S8 Fig Phenotype of cycloheximide-treated seedlings. WT plants were grown on top of a sterile disc of synthetic fabric for 7 days. After transferring the disc with the seedlings to fresh medium supplemented with 100 μM cycloheximide, pictures of the same individual were taken at the indicated times.
(PDF)

S9 Fig Immunoblot analyses of DXS, Hsp70 and Hsp100 chaperones in the indicated amounts of extracts from 7-day-old WT seedlings. (PDF)

S1 Table List of mutants used in this work. (PDF)
