# Correction: Specific Hsp100 Chaperones Determine the Fate of the First Enzyme of the Plastidial Isoprenoid Pathway for Either Refolding or Degradation by the Stromal Clp Protease in Arabidopsis

**DOI:** 10.1371/journal.pgen.1010216

**Published:** 2022-05-09

**Authors:** Pablo Pulido, Ernesto Llamas, Briardo Llorente, Salvador Ventura, Louwrance P. Wright, Manuel Rodríguez-Concepción

The following information is missing from the Funding statement: This work was funded by grants from MINECO and FEDER (BIO2015-71703-REDT and BIO2014-59092-P) and Generalitat de Catalunya (2014SGR-1434). We also acknowledge the financial support from the Severo Ochoa Programme for Centres of Excellence in R&D 2016–2019 (SEV-2015-0533).
