# Supplementary material for: Specific Hsp100 Chaperones Determine the Fate of the First Enzyme of the Plastidial Isoprenoid Pathway for Either Refolding or Degradation by the Stromal Clp Protease in Arabidopsis
Source: PLoS Genet. 2016 Jan 27;12(1):e1005824. doi: 10.1371/journal.pgen.1005824 (PMC4729485; doi:10.1371/journal.pgen.1005824)
Supplement: S1 Fig — (PDF) [file pgen.1005824.s001.pdf]

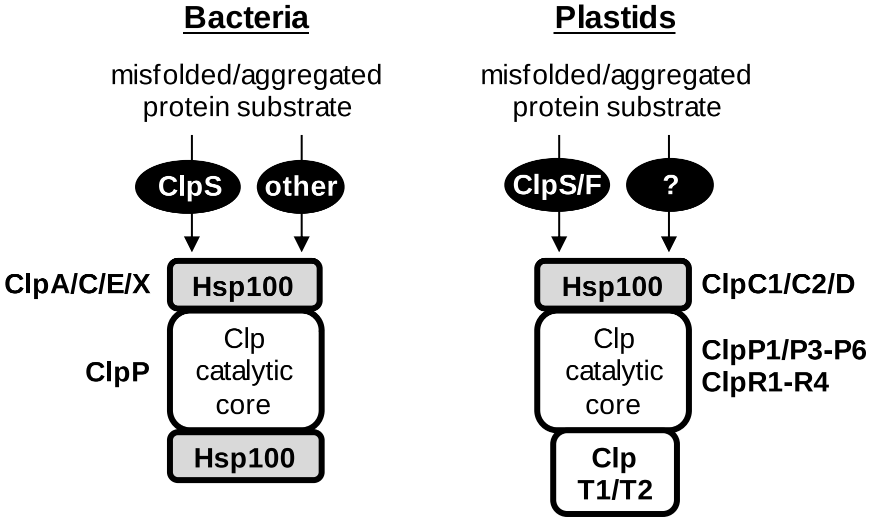
Bacteria
misfolded/aggregated protein substrate
ClpS
other
ClpA/C/E/X
Hsp100
ClpP
Clp catalytic core
Hsp100
Plastids
misfolded/aggregated protein substrate
ClpS/F
?
Hsp100
ClpC1/C2/D
Clp catalytic core
ClpP1/P3-P6
ClpR1-R4
Clp T1/T2
