# Supplementary figures and images for: Specific Hsp100 Chaperones Determine the Fate of the First Enzyme of the Plastidial Isoprenoid Pathway for Either Refolding or Degradation by the Stromal Clp Protease in Arabidopsis
Source: PLoS Genet. 2016 Jan 27;12(1):e1005824. doi: 10.1371/journal.pgen.1005824 (PMC4729485; doi:10.1371/journal.pgen.1005824)

WT

*clpc1-1**clpc1-2**clpc2-1**clpc2-2* $\alpha$ DXS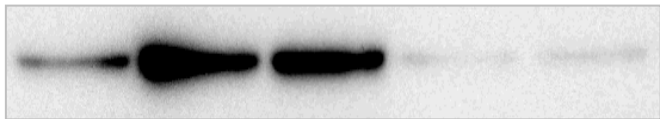 $\alpha$  Hsp70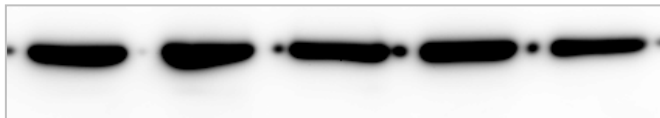 $\alpha$  ClpC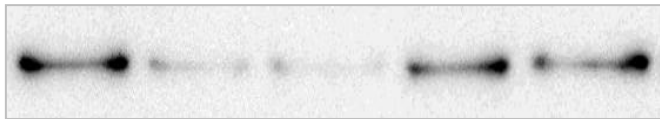 $\alpha$  ClpB3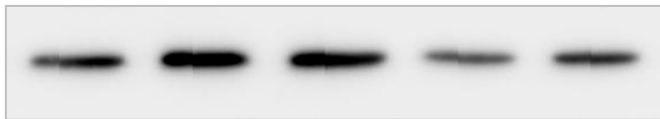

LC

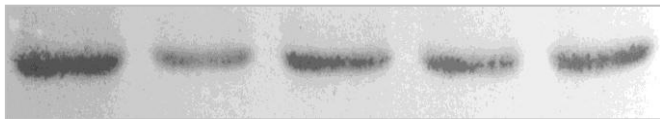

Supplement: S2 Fig — LC, loading control. (PDF) [file pgen.1005824.s002.pdf]

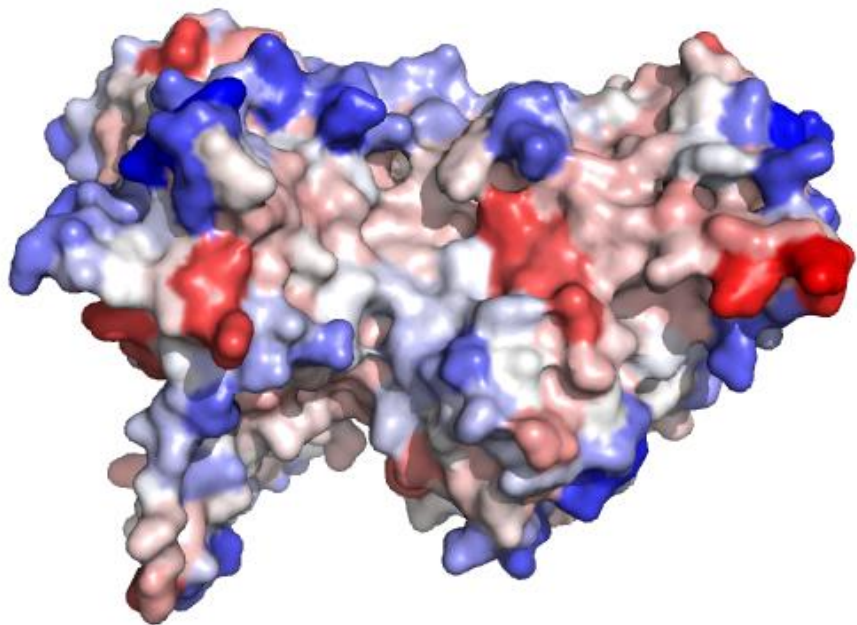

Supplement: S3 Fig — The protein surface is colored according to Aggrescan3D score in gradient from red (high predicted aggregation propensity) to white (negligible impact on protein aggregation) to blue (high predicted solubility). The figure was generated with PyMOL using the aggregation propensities encoded in the temperature factor column of the A3D.pdb output. (PDF) [file pgen.1005824.s003.pdf]

**A***35S:DXS-GFP*

WT

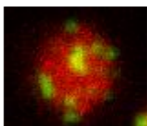*j20*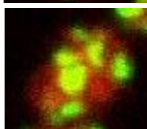**B**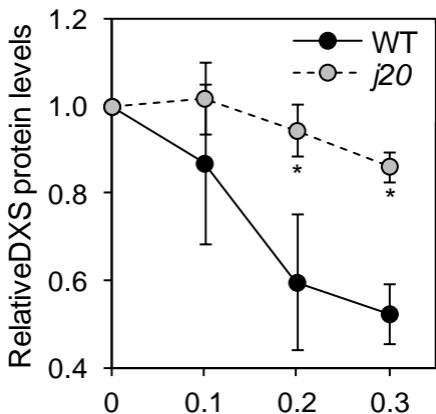

WT

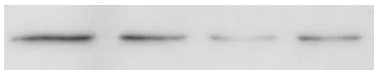*j20*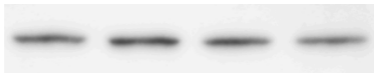

Supplement: S4 Fig — (A) Confocal microscopy analysis of GFP (green) and chlorophyll (red) fluorescence distribution within the chloroplast. Representative images obtained with the same confocal parameters from siblings harboring the same T-DNA insertion with the 35S:DXS-GFP construct in a wild-type (WT) or J20-defective (j20) background are shown. (B) Analysis of DXS protein abundance by immunoblot analysis of protein extracts from WT and j20 plants incubated with the indicated concentrations (μg/ml) of proteinase K. Representative blots and quantitative data corresponding to the mean and SE values of n = 4 independent experiments are shown. Asterisks mark statistically significant differences (t test: p<0.05) relative to WT samples. (PDF) [file pgen.1005824.s004.pdf]

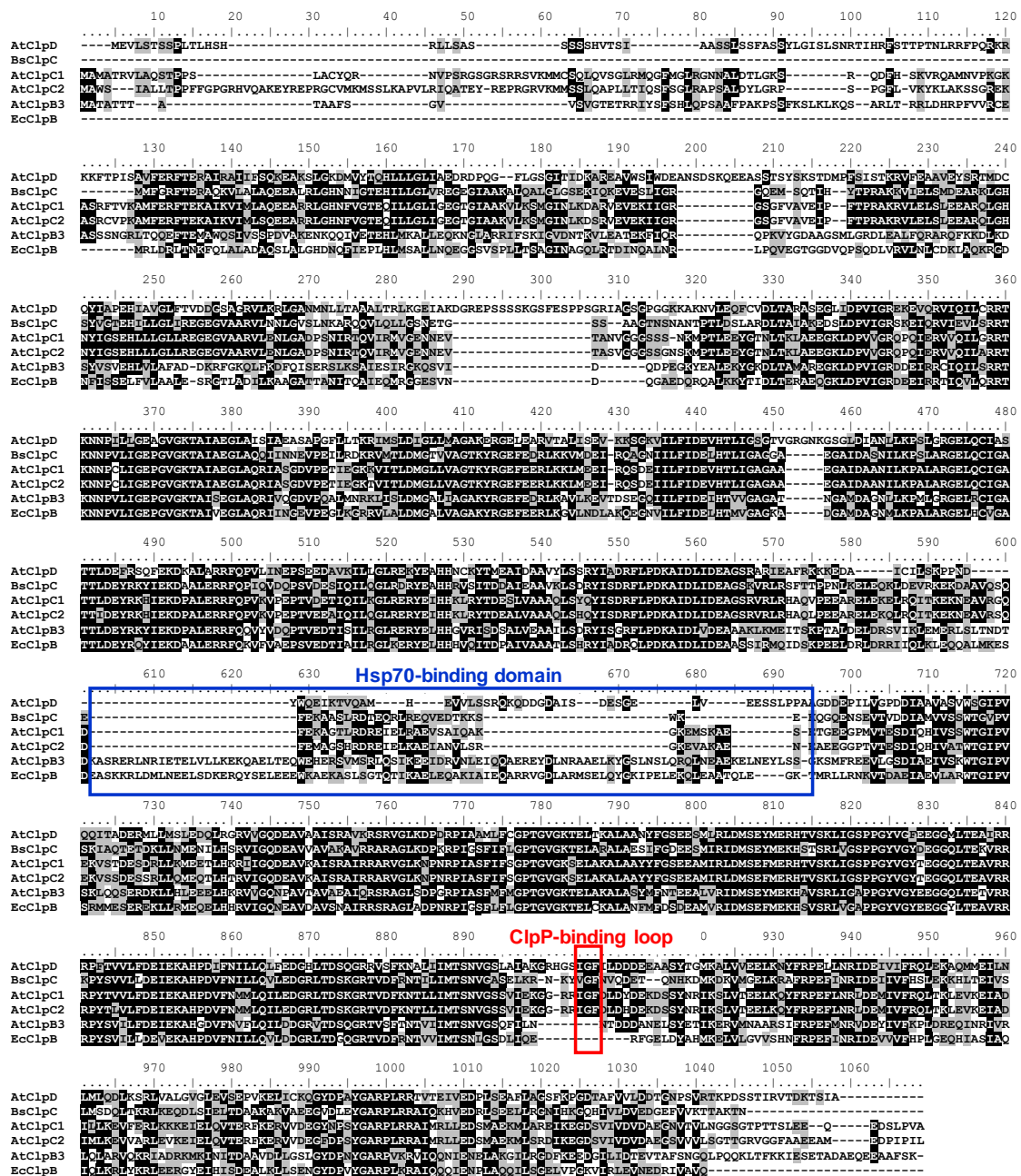

Supplement: S5 Fig — Alignment was performed using Clustal Omega (www.ebi.ac.uk/Tools/msa/clustalo) with protein sequences of Arabidopsis thaliana ClpD (At5g51070), ClpC1 (At5g50920), ClpC2 (At3g48870), and ClpB3 (At5g15450), Bacillus subtilis ClpC (AAA19233), and Escherichia coli ClpB (EDV64786). The generated alignment was then edited with Bioedit (http://www.mbio.ncsu.edu/bioedit/page2.html). The domain responsible for the interaction of E. coli ClpB with Hsp70 is boxed in blue. The tripeptide loop shown to be required for interaction with ClpP subunits of the Clp protease complex is boxed in red. (PDF) [file pgen.1005824.s005.pdf]

CLM concentration ( $\mu\text{M}$ )

0

2

4

WT

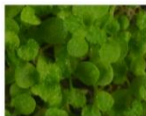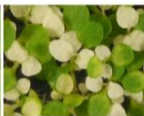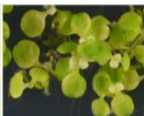

*j20*

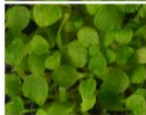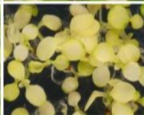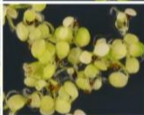

*clpc1*

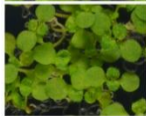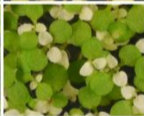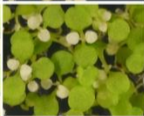

*j20 clpc1*

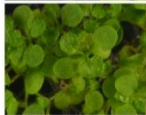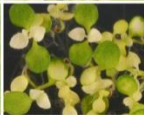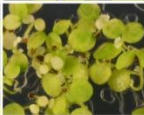

Supplement: S6 Fig — Representative pictures of plants of WT, single mutant, and double mutant lines germinated and grown for 10 days in the presence of the indicated concentrations of CLM are shown. (PDF) [file pgen.1005824.s006.pdf]

Time (h)

0

3

6

9

12

100 $\mu$ M  
cycloheximide

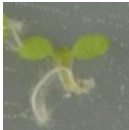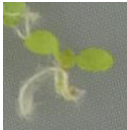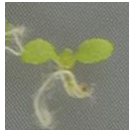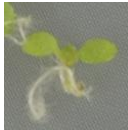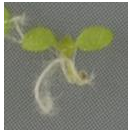

Supplement: S8 Fig — WT plants were grown on top of a sterile disc of synthetic fabric for 7 days. After transferring the disc with the seedlings to fresh medium supplemented with 100 μM cycloheximide, pictures of the same individual were taken at the indicated times. (PDF) [file pgen.1005824.s008.pdf]

Total protein ( $\mu\text{g}$ )

10

20

30

40

$\alpha\text{DXS}$

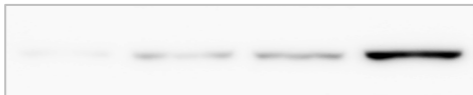

$\alpha\text{ClpB3}$

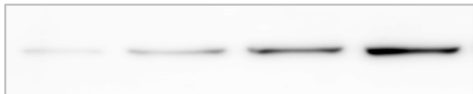

2.5

5

10

20

$\alpha\text{Hsp70}$

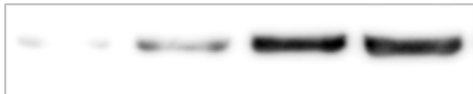

$\alpha\text{ClpC}$

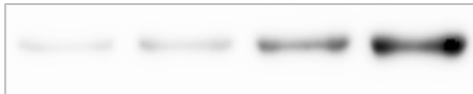

Supplement: S9 Fig — (PDF) [file pgen.1005824.s009.pdf]
