# Supplementary material for: Specific Hsp100 Chaperones Determine the Fate of the First Enzyme of the Plastidial Isoprenoid Pathway for Either Refolding or Degradation by the Stromal Clp Protease in Arabidopsis
Source: PLoS Genet. 2016 Jan 27;12(1):e1005824. doi: 10.1371/journal.pgen.1005824 (PMC4729485; doi:10.1371/journal.pgen.1005824)
Supplement: S7 Fig — Data were obtained from the Arabidopsis eFP browser at www.bar.utoront.ca and correspond to the gene expression map of Arabidopsis development (A) and abiotic stress treatments (B). (PDF) [file pgen.1005824.s007.pdf]

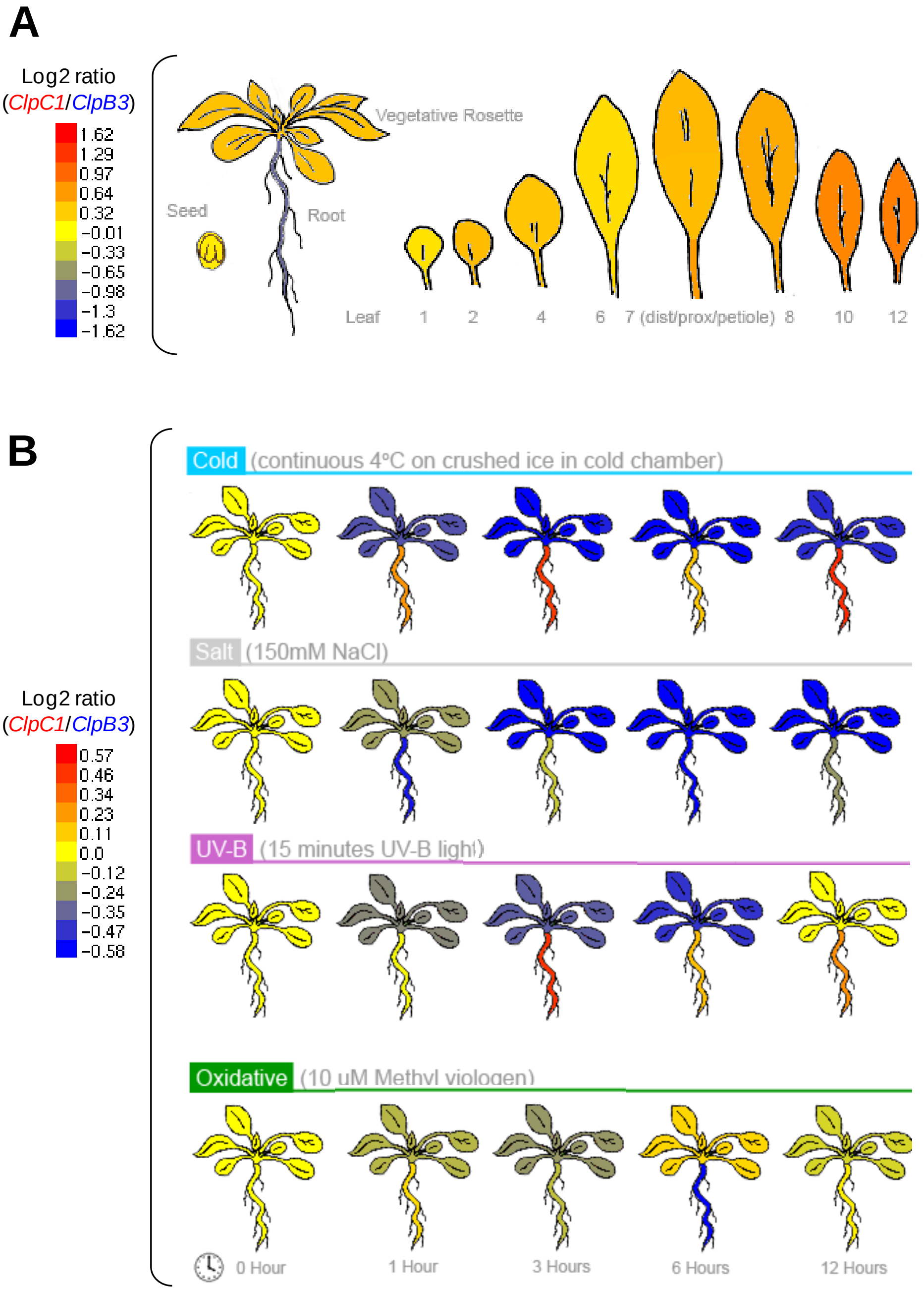
A
Log2 ratio
(ClpC1/ClpB3)
1.62
1.29
0.97
0.64
0.32
−0.01
−0.33
−0.65
−0.98
−1.3
−1.62
Vegetative Rosette
Seed
Root
Leaf 1 2 4 6 7 (dist/prox/petiole) 8 10 12
B
Log2 ratio
(ClpC1/ClpB3)
0.57
0.46
0.34
0.23
0.11
0.0
−0.12
−0.24
−0.35
−0.47
−0.58
Cold (continuous 4°C on crushed ice in cold chamber)
Salt (150mM NaCl)
UV-B (15 minutes UV-B light)
Oxidative (10 uM Methyl viologen)
0 Hour
1 Hour
3 Hours
6 Hours
12 Hours
