# Supplementary material for: Specific Hsp100 Chaperones Determine the Fate of the First Enzyme of the Plastidial Isoprenoid Pathway for Either Refolding or Degradation by the Stromal Clp Protease in Arabidopsis
Source: PLoS Genet. 2016 Jan 27;12(1):e1005824. doi: 10.1371/journal.pgen.1005824 (PMC4729485; doi:10.1371/journal.pgen.1005824)
Supplement: S1 Table — (PDF) [file pgen.1005824.s010.pdf]

Table S1. List of mutants used in this work. References associated to this table are indicated below.

Protein	Other names	AGI locus	Mutant line/type	Allele	Reference
**ClpB3**	APG6	AT5G15450	SALK_111575	*clpb3-2*	(1)
**ClpD**	ERD1	AT5G51070	SALK_110649	*clpd*	This work
**ClpC1**	Hsp93-V	AT5G50920	SALK_014058	*clpc1-1* (*hsp93-V-2*)	(2, 3)
			SAIL_873_G11	*clpc1, clpc1-2* (*hsp93-V-1*)	(2, 3)
**ClpC2**	Hsp93-III	AT3G48870	SAIL_ 622_B05	*clpc2-1* (*hsp93-III-1*)	(4)
			GK_039E12	*clpc2, clpc2-2* (*hsp93-III-2*)	(4)
**ClpR1**	SVR2	AT1G49970	SALK_088407	*clpr1-2*	(5)
**ClpS**	ClpS1/ClpT	AT1G68660	SAIL_326b	*clps*	(6)
**ClpT1**	ClpS1	AT4G25370	SALK_052772	*clpt1*	(7)
**ClpT2**	ClpS2	AT4G12060	SALK_132943	*clpt2-2*	This work
**Deg1**	DegP1	AT3G27925	RNAi	*deg1-4*	(8)
**Deg2**	DegP2	AT2G47940	SALK_115784	*deg2-1*	(9)
**Deg5**	DegP5	AT4G18370	SALK-099162	*deg5*	(10)
**Deg7**	DegP7	AT3G03380	SALK_075584	*deg7*	(11)
**Deg8**	DegP8	AT5G39830	SALK-004770	*deg8*	(10)
**FtsH1**		AT1G50250	T-DNA	*ftsh1*	(12)
**FtsH2**	Var2	AT2G30950	EMS	*var2*	(13)
**FtsH5**	Var1	AT5G42270	EMS	*var1*	(14)
**FtsH8**		AT1G06430	T-DNA	*ftsh8*	(12)
**cpHsp70.2**	Hsp70-7	AT5G49910	SALK_095715	*hsp70.2*	(15)
**J20**	DJC26	AT4G13830	SAIL_1179_E04	*j20-1*	(16)
**Lon1**		AT5G26860	SALK_012797	*lon1-2*	(17)
**Lon4**		AT3G05790	SALK_120414	*lon4*	This work
**DXS**	CLA1	AT4G15560	T-DNA	*dxs-1 (cla1)*	(18, 19)
**HDR**	IDS, LytB, IspH, CLB6	AT4G34350	SALK_026807	*hdr-3*	(20)

1. Lee, U., Rioflorido, I., Hong, S.W., Larkindale, J., Waters, E.R., and Vierling, E. (2007). The Arabidopsis ClpB/Hsp100 family of proteins: chaperones for stress and chloroplast development. Plant J 49, 115-127.
2. Kovacheva, S., Bedard, J., Patel, R., Dudley, P., Twell, D., Rios, G., Koncz, C., and Jarvis, P. (2005). In vivo studies on the roles of Tic110, Tic40 and Hsp93 during chloroplast protein import. Plant J 41, 412-428.
3. Sjögren, L.L., MacDonald, T.M., Sutinen, S., and Clarke, A.K. (2004). Inactivation of the clpC1 gene encoding a chloroplast Hsp100 molecular chaperone causes growth retardation, leaf chlorosis, lower photosynthetic activity, and a specific reduction in photosystem content. Plant Physiol 136, 4114-4126.
4. Kovacheva, S., Bedard, J., Wardle, A., Patel, R., and Jarvis, P. (2007). Further in vivo studies on the role of the molecular chaperone, Hsp93, in plastid protein import. Plant J 50, 364-379.

5. Flores-Pérez, U., Sauret-Güeto, S., Gas, E., Jarvis, P., and Rodríguez-Concepción, M. (2008). A mutant impaired in the production of plastome-encoded proteins uncovers a mechanism for the homeostasis of isoprenoid biosynthetic enzymes in Arabidopsis plastids. Plant Cell 20, 1303-1315.
6. Nishimura, K., Asakura, Y., Friso, G., Kim, J., Oh, S.H., Rutschow, H., Ponnala, L., and van Wijk, K.J. (2013). ClpS1 is a conserved substrate selector for the chloroplast Clp protease system in Arabidopsis. Plant Cell 25, 2276-2301.
7. Sjogren, L.L., and Clarke, A.K. (2011). Assembly of the chloroplast ATP-dependent Clp protease in Arabidopsis is regulated by the ClpT accessory proteins. Plant Cell 23, 322-332.
8. Sun, X., Ouyang, M., Guo, J., Ma, J., Lu, C., Adam, Z., and Zhang, L. (2010). The thylakoid protease Deg1 is involved in photosystem-II assembly in Arabidopsis thaliana. Plant J 62, 240-249.
9. Huesgen, P.F., Schuhmann, H., Adamska, I. (2006). Photodamaged D1 protein is degraded in Arabidopsis mutants lacking the Deg2 protease. FEBS Lett 580, 6929-6932.
10. Sun, X., Peng, L., Guo, J., Chi, W., Ma, J., Lu, C., and Zhang, L. (2007). Formation of DEG5 and DEG8 complexes and their involvement in the degradation of photodamaged photosystem II reaction center D1 protein in Arabidopsis. Plant Cell 19, 1347-1361.
11. Sun, X., Fu, T., Chen, N., Guo, J., Ma, J., Zou, M., Lu, C., and Zhang, L. (2010). The stromal chloroplast Deg7 protease participates in the repair of photosystem II after photoinhibition in Arabidopsis. Plant Physiol 152, 1263-1273.
12. Sakamoto, W., Zaltsman, A., Adam, Z., and Takahashi, Y. (2003). Coordinated regulation and complex formation of yellow variegated1 and yellow variegated2, chloroplastic FtsH metalloproteases involved in the repair cycle of photosystem II in Arabidopsis thylakoid membranes. Plant Cell 15, 2843-2855.
13. Takechi, K., Sodmergen, Murata, M., Motoyoshi, F., and Sakamoto, W. (2000). The YELLOW VARIEGATED (VAR2) locus encodes a homologue of FtsH, an ATP-dependent protease in Arabidopsis. Plant Cell Physiol 41, 1334-1346.
14. Sakamoto, W., Tamura, T., Hanba-Tomita, Y., and Murata, M. (2002). The VAR1 locus of Arabidopsis encodes a chloroplastic FtsH and is responsible for leaf variegation in the mutant alleles. Genes Cells 7, 769-780.
15. Su, P.H., and Li, H.M. (2008). Arabidopsis stromal 70-kD heat shock proteins are essential for plant development and important for thermotolerance of germinating seeds. Plant Physiol 146, 1231-1241.
16. Pulido, P., Toledo-Ortiz, G., Phillips, M.A., Wright, L.P., and Rodriguez-Concepcion, M. (2013). Arabidopsis J-protein J20 delivers the first enzyme of the plastidial isoprenoid pathway to protein quality control. Plant Cell 25, 4183-4194.
17. Rigas, S., Daras, G., Laxa, M., Marathias, N., Fasseas, C., Sweetlove, L.J., and Hatzopoulos, P. (2009). Role of Lon1 protease in post-germinative growth and maintenance of mitochondrial function in Arabidopsis thaliana. New Phytol 181, 588-600.
18. Mandel, M.A., Feldmann, K.A., Herrera-Estrella, L., Rocha-Sosa, M., and Leon, P. (1996). CLA1, a novel gene required for chloroplast development, is highly conserved in evolution. Plant J 9, 649-658.
19. Phillips, M.A., Leon, P., Boronat, A., and Rodriguez-Concepcion, M. (2008). The plastidial MEP pathway: unified nomenclature and resources. Trends Plant Sci 13, 619-623.
20. Pokhilko, A., Bou-Torrent, J., Pulido, P., Rodriguez-Concepcion, M., and Ebenhoh, O. (2015). Mathematical modelling of the diurnal regulation of the MEP pathway in Arabidopsis. New Phytol 206, 1075–1085.
